# Examining the Feasibility, Acceptability, and Preliminary Efficacy of an Immersive Virtual Reality–Assisted Lower Limb Strength Training for Knee Osteoarthritis: Mixed Methods Pilot Randomized Controlled Trial

**DOI:** 10.2196/52563

**Published:** 2024-09-27

**Authors:** Hermione Hin Man Lo, Marques Ng, Pak Yiu Hugo Fong, Harmony Hoi Ki Lai, Bo Wang, Samuel Yeung-shan Wong, Regina Wing Shan Sit

**Affiliations:** 1 The Nethersole School of Nursing, The Chinese University of Hong Kong, Hong Kong, Hong Kong (China); 2 Jockey Club School of Public Health and Primary Care, The Chinese University of Hong Kong, Room 209, School of Public Health Building, Prince of Wales Hospital, Ngan Shing Street, Shatin, New Territories, Hong Kong, 999077, Hong Kong (China), 852 22528452, 852 26063500

**Keywords:** virtual reality, VR, immersive, knee, joint, arthritis, arthritic, osteoarthritis, knee osteoarthritis, gerontology, geriatric, older adult, elder, elderly, older person, older people, aging, pain, function, acceptability, user experience, RCT, randomized, controlled trial, limb, strength, muscle, muscular, physiotherapy

## Abstract

**Background:**

Knee osteoarthritis prevalently causes significant pain, activity limitations, psychological distress, and reduced quality of life. Despite lower limb strength training being a core treatment for knee osteoarthritis, adherence remains a challenge, prompting the exploration of virtual reality (VR) to improve exercise compliance. Recent research suggests the potential of VR in providing enhanced pain management and functional outcomes for knee osteoarthritis, necessitating further exploration of immersive VR technology.

**Objective:**

We aimed to study the feasibility, acceptability, and preliminary efficacy of an immersive VR-assisted lower limb strength training for knee osteoarthritis (VRiKnee).

**Methods:**

A convergent, parallel, mixed methods study was conducted in 30 participants with knee osteoarthritis. After 1:1 randomization, the VRiKnee group (n=15) was assigned to perform repetitive concentric quadriceps and isometric vastus medialis oblique exercise in an immersive environment using a head-mounted display for 12 weeks. The control group (n=15) completed the same exercises without VRiKnee. VRiKnee participants were interviewed at week 12 to study VRiKnee acceptability and user experience. Quantitative data included feasibility outcomes such as recruitment, dropout, and exercise adherence rates, and effectiveness outcomes such as the numeric rating scale, the Western Ontario and McMaster Universities Osteoarthritis Index (100 points) pain and function subscales, and objective physical activity measured by metabolic equivalents of task using an ActivPAL accelerometer. Qualitative data were analyzed by thematic analysis, followed by integration with quantitative data using joint displays.

**Results:**

The recruitment rate was 100% (N=30), with enrollment of 30 participants in 7.57 weeks. The median age was 63.5 (IQR 61.8‐66.3) years, with 76% (n=23) being female. The response rates were 80% and 93% for the VRiKnee and control groups. Dropout rates were 13% for VRiKnee and 7% for the control group. Median exercise adherence was 77% (IQR 37-104%) for VRiKnee and 62% (IQR 40-166%) for the control group, respectively, with adherence reduction over this study’s period and no significant intergroup differences (*P*=.82). No statistically significant differences were observed in primary and secondary outcomes, though positive trends were observed in pain and stiffness. Cybersickness was reported by 5 (33%) participants in the VRiKnee group. In the qualitative analysis, 4 themes, 11 subthemes, and 16 quotes were generated, identifying facilitators and barriers with practical suggestions to enhance the usability of VRiKnee.

**Conclusions:**

VRiKnee demonstrated feasibility, acceptability, and potential efficacy in managing knee osteoarthritis. Future trials of larger sample sizes and better VR designs will confirm its role in clinical practice.

## Introduction

Knee osteoarthritis is a common degenerative joint disease with a global prevalence of 22.9% in participants aged 40 years and older [1]. It is a major contributor to disability worldwide, causing considerable pain, activity limitations, psychological distress, and reduced quality of life for those affected [2-4]. According to Osteoarthritis Research Society International in 2019, land-based exercise is a core treatment for knee osteoarthritis, for which lower limbs strength training is often recommended [5-7]. In clinical practice, exercise programs often involve initial supervision by a clinician, followed by unsupervised home exercise. Ideally, regular participation in exercise should be one of the long-term goals for knee osteoarthritis; unfortunately, adherence to home exercise is often poor [8]. Therefore, strategies are needed to improve adherence to home exercise.

Recently, studies have suggested that technology advancements may increase attractiveness of exercise programs, thus further improving its compliance, adherence, and clinical outcomes [9,10]. In this context, innovative approaches, such as virtual reality (VR), have gained attention as potential interventions to enhance exercise adherence and outcomes for participants with knee osteoarthritis. VR is a digital technology that incorporates the use of interactive simulations created with computer hardware and software to present users with opportunities to engage in environments that appear and feel similar to real-world objects and events [11,12]. Within VR applications, an important distinction can be made between immersive and nonimmersive media, which differs in spatial presences [13]. With immersive technology, participants view the full panorama and are essentially inside the created environment. In a nonimmersive environment, virtual content is based on how the device (PC, smartphone, or tablet) is moved or rotated, and participants are only external observers.

An increasing number of trials have demonstrated the positive role of VR-assisted interventions in chronic pain management [14]. VR distracts users from their noxious pain perceptions by shifting their focus into video games, thus increasing their pain tolerance [15,16]. The gaming elements further enhance users’ performances and motivation to exercise [17,18]. VR also facilitates skill-building for regulating painful stimuli through stimulating visual, auditory, and proprioception senses [16]. Numerous studies have supported VR-assisted physical therapy in reducing pain and improving function in low back pain and neck pain [19-21]. However, little is known about the effectiveness of VR-assisted physical therapy for knee osteoarthritis. To the best of our knowledge, only 3 trials evaluated nonimmersive VR-assisted balance and proprioception training, and findings suggested its benefits in reducing pain and improving function in knee osteoarthritis [22-24]. Since evidence has highlighted that the exposure to an immersive VR is able to elicit a better sense of presence and potentially impact the effectiveness of VR treatments [25], it is worth to explore immersive VR-assisted physical therapy in the rehabilitation of knee osteoarthritis.

This study aimed to pilot-test the feasibility, acceptability, and preliminary efficacy of an immersive VR-assisted lower limb strength training for knee osteoarthritis (VRiKnee) through a mixed methods approach. We hypothesized that VRiKnee was feasible as a home-based exercise for knee osteoarthritis. The quantitative measures would provide insights into the trend of clinical effectiveness, guiding the design of larger-scale trials, whereas the qualitative measures would uncover potential barriers, enabling the enhancement of future VR interventions.

## Methods

### Study Design

A convergent, parallel, mixed method study was used to gain an in-depth understanding of feasibility and acceptability of applying immersive VR on our selected population [26]. Quantitative and qualitative data were conducted concurrently in a 12-week, 2-arm, pilot randomized controlled trial design (CHiCTR2100046313). This study was conducted in Hong Kong from June 1, 2021, to March 18, 2022, in 3 separate batches. All collected quantitative and qualitative data were equally weighted, independently analyzed, and then integrated to generate results [26].

### Ethical Considerations

This study complied with the Declaration of Helsinki, and ethical approval was obtained from the Joint Chinese University of Hong Kong–New Territories East Cluster Clinical Research Ethics Committee (2021.052). Written informed consent was obtained from all participants. All data were deidentified and kept confidential and were disposed on completion of this study.

### Settings and Participants

Participants were recruited from an existing community project titled “CUHK-Jockey Club Pain Relief Project for Seniors,” a charity program that offered nonpharmacological pain management to older adults with chronic musculoskeletal pain [27]. The eligibility criteria were screened by a trained research assistant and confirmed by a primary care physician. The inclusion criteria were a diagnosis of knee osteoarthritis based on clinical and radiographic criteria, as defined by the American Rheumatology College; moderate to severe knee pain for at least 3 months; pain intensity score ≥4 on a numeric rating scale (NRS) of 10; and use of a smartphone [28]. The exclusion criteria were decompensated organic and psychiatric disease; contraindications to VR therapy due to history of epilepsy or severe myopia (>−3.5 diopters); and comorbidities that may impede active participation in this study. Participants were recruited in 3 separate batches based on the availability of lower limb sensors.

### Interventions

All participants attended a 30-minute health talk led by a registered physiotherapist to explain and demonstrate the home-based exercises. Further, 2 sets of lower limbs strengthening exercise were selected from the Ottawa panel clinical practice guideline and validated by a physiotherapist and a primary care physician [29]. This included repeated knee-extension exercise for concentric quadriceps training and squeezing a fitness ring between the thighs for vastus medialis oblique isometric training, both to be performed in a sitting position.

Participants in the VRiKnee group were instructed to perform lower limb exercises for 12 weeks using an immersive VR platform developed by our team. A smartphone app (iOS or Android) which captured outdoor garden scenes was used to create an immersive environment and participants were immersed using a head-mounted display (HMD) device (VR Shinecon 5.0). The app provided a virtual environment with ambient audio, an amateur as a coach, and interactive animations with visual feedback that encourage practice, that is, virtual flowers that would blossom with successful exercise moves and a timer for unlocking the 3 levels of difficulty. The app also recorded participants’ accumulated moves, total exercise time, and game level in a virtual scoreboard (Multimedia Appendix 1).

Participants in the control group were instructed to perform the same set of exercises as guided by paper-based education pamphlets. Both groups were advised to perform the exercise 5 days per week, with an expected duration of 30 min/d for 12 weeks. An exercise diary was given to the control group to record exercise participation.

### Sample Size Calculation

We applied the stepped rule of thumb in this pilot sample size calculation [30]. With a proposed future main trial design of 90% power and 2-sided 5% significance, a total of 30 participants (15 at each arm) would be able to detect an assumed effect size of 0.5.

### Randomization, Allocation, Concealment, and Blinding

An off-site statistician performed 1:1 randomization using Random Allocation Software (version 1.0) to allocate and control groups. The allocation sequence was concealed from investigators and participants using sequentially numbered, opaque, and sealed envelopes. The corresponding envelopes were opened at the time of intervention assignment after all the enrolled participants had undergone all baseline assessments. It was not possible to blind participants and research assistants implementing interventions in this open-label study; however, data collectors and outcome adjudicators were blinded to the allocation status.

### Data Collection

The outcome measures were collected through face-to-face interviews at baseline, 6 weeks, and 12 weeks. Demographic data such as age, gender, the number, and type of comorbid illnesses were recorded.

### Quantitative Outcomes

#### Primary Outcomes

The primary outcome was to assess the feasibility of VRiKnee for knee osteoarthritis. Assessments included recruitment rate, dropout rate, response rate, intervention adherence rate, and adverse events. Recruitment rate was defined by the proportion of eligible participants who successfully enrolled into this study. Response rate was the percentage of usable responses obtained from our quantitative data questionnaires, calculated over the number of eligible participants. Dropout rate was the proportion of participants who dropped out of this study before its completion. Intervention adherence rate was measured by the Timeline Followback for Exercise, which is a validated retrospective self-reported calendar that documents exercise participation [31]. It was calculated by the total self-reported exercise time over the expected time spent on lower limb strengthening exercises in this study’s period, that is, total exercise time (min) in wk/(30 min per d × 5 d per wk × number of wk). Adverse events or side effects were collected by participants’ exercise records at each visit. Success criteria for this pilot study were set with prespecified thresholds of a 60% recruitment rate, a 70% response rate, and a <20% dropout rate.

#### Secondary Outcomes

Secondary outcomes evaluated the treatment effect at 12 weeks. These included pain intensity, physical function, and health-related quality of life. Pain intensity was measured using the NRS (0‐10) [32] and the Western Ontario and McMaster Universities Osteoarthritis Index (WOMAC) pain subscale [33,34]. Physical function was measured subjectively by the WOMAC function subscale [33,34] and objectively by the number of steps captured by ActivPAL monitor (PAL Technologies Ltd). ActivPAL is a thigh-worn accelerometer that distinguishes body posture and movement and provides objective exercise participation measurements [35,36]. Participants were assigned to wear the ActivPAL at their anterior upper thigh for 7 consecutive days at baseline and at 12 weeks, respectively. All ActivPAL data that recorded at least 4 days of activities over 20 hours were considered as valid [37,38]. The metabolic equivalents of task (MET) equation was used to measure the level of physical activities [39]. Health-related quality of life was measured by the EuroQol-5D [40,41].

### Qualitative Outcomes

A concurrent approach was used to understand the acceptability toward VRiKnee. All participants were recruited to focus group interviews at 12-week using convenience sampling and qualitative data was collected until theoretical saturation [42]. A licensed counseling assistant (HHKL) with experience in qualitative interviews and a registered nurse (HHML) held the semistructured interviews with open-ended questions (Multimedia Appendix 2) to assess user experience and technology acceptance [43,44]. To engage participants in a dialogue, follow-up questions were asked according to individual responses. Since this study was conducted during the COVID-19 pandemic, both face-to-face and Zoom (Zoom Video Communications, Qumu Corporation) videoconferencing were used for focus group interviews [45].

### Statistical Methods and Analysis

Quantitative data were statistically analyzed with IBM SPSS Statistics software (version 28.0.1.1, IBM Corp). Between-group differences were compared with the Mann-Whitney test for continuous variables and the Fisher exact test for categorical variables [46]. Prior to the intention-to-treat (ITT) analysis, missing values were imputed using a Markov chain Monte Carlo model, in which 10 completed data sets were imputed under the assumption that data were missing at random [47-49]. Imputed dependent variables included the NRS, WOMAC, EQ-5D, and MET; imputed independent variables or covariates included gender, number of chronic diseases, and baseline MET. The parameters were combined according to the Rubin rule, and a linear mixed model was used to analyze results. Analysis of covariance was used to assess the intervention effects on secondary outcomes at week 6 and 12 following the ITT analysis, adjusting baseline measurements at randomization [50].

Qualitative data were collected and analyzed with our quantitative data. Each semistructured interview was transcribed verbatim and analyzed using thematic analysis [51]. Further, 2 researchers (HHML and HHKL) read the transcripts line by line to familiarize with the data and initial codes were then formulated. The coding structure was then validated by 2 authors (MN and RWSS) independently. Codes sharing similar meanings were consolidated and organized into potential themes. Thematic maps were generated for review of themes in relation to the coded extract [52]. Similar codes were grouped into categories and themes and were discussed within the research team until consensus was met. We initiated coding and analysis of data early after batch one to identify possible patterns and themes, and qualitative data was collected until theoretical saturation was reached [53]. The researcher reflexivity of this study was enhanced with several strategies [54]. First, the interviews were led by HHKL, a counseling assistant with experience in qualitative interviews; HHML is a registered nurse with experience in older adult care, who participated in observation note taking during the interviews. Second, a reflexive journal was kept throughout the research process to increase transparency. To minimize individual biases, reflexive team discussions were carried out among the research team throughout the interpretation phase. The work was supported by RWSS, a family medicine physician with clinical and research experience, and MN who is an expert on mixed method studies.

Finally, data-mixing occurred when both quantitative and qualitative data were analyzed using side-by-side comparisons and joint displays [55].

## Results

### Overview

In total, 30 participants with a median age of 63.5 (IQR 61.8‐66.3) years, 76% (n=23) of whom were female, were recruited. No statistical differences were found between the groups in terms of baseline measurements of variables (all *P* values were >.05). Participants’ baseline characteristics are summarized in Table 1.

**Table 1.** Participants’ baseline characteristics.

Demographic data	Total (N=30)	VR^a^ group (n=15)	Control group (n=15)	*P* value^b^
Age (years), median (IQR)	63.5 (61.8‐66.3)	63 (60‐67)	64 (62‐65)	.97
Gender (female), n (%)	23 (76.7)	10 (66.7)	13 (86.7)	.39
Retired, n (%)	23 (76.7)	12 (80)	11 (70.3)	>.99
Number of comorbidities^c^, median (IQR)	2 (0‐2.3)	2 (1.5‐2.5)	1 (0‐2)	.22
BMI (kg/m^2^), median (IQR)	22.8 (21‐25.7)	23.8 (21.3‐26.6)	21.6 (20.9‐24.4)	.17
NRS^d^ (score: range 0‐10), median (IQR)	5.5 (4-7)	6 (5‐7)	5 (3‐6)	.11
WOMAC^e^ (score: range 0‐2400), median (IQR)	703 (424.3‐1231.8)	889 (464‐1150)	464.5 (284‐1252)	.33
WOMAC pain (score: range 0‐500), median (IQR)	142.5 (95.8‐250)	150 (112‐240)	120 (67‐271)	.35
WOMAC stiffness (score: range 0‐200), median (IQR)	71 (35.3‐117.8)	99 (54‐113)	47 (17‐132)	.12
WOMAC function (score: range 0‐1700), median (IQR)	478 (273.5‐829.5)	527 (320‐828)	377 (170‐834)	.33
EQ-VAS^f^, median (IQR)	70 (57.5‐76.3)	65 (50‐80)	70 (60‐70)	.57
Metabolic equivalents of task^g^, median (IQR)	34.7 (33.5‐35.4)	34.1 (33.2‐35.2)	34.9 (33.9‐35.6)	.20

a VR: virtual reality.

b Fisher exact test was used for categorical variables . Mann-Whitney test was used for nonnormal continuous variables.

c Participants reported comorbidities including hypertension, diabetes mellitus, dyslipidemia, cerebrovascular disease, indigestion, cataract, hyperthyroidism or hypothyroidism, tinnitus, fatty liver, gall stones, indigestion, constipation, gastritis, cataract, cerebrovascular disease, and cancer.

d NRS: numeric rating scale.

e WOMAC: Western Ontario and McMaster Universities Osteoarthritis Index.

f EQ-VAS: Euro-Qol-visual analogue scale.

g One invalid ActivPAL record due to device failure from control group.

### Primary Outcomes

Recruitment was conducted between May 27, 2022, to November 3, 2022, in 3 separated batches; the total recruitment time was 7.57 weeks. A total of 100 registrations were received; 29 did not have knee osteoarthritis, 30 did not have smartphones that operate on Android or iOS, and 11 had comorbidities that precluded their participation. The remaining 30 eligible participants were all enrolled to this study and successfully randomized, with a recruitment rate of 100%. The response rates were 80% and 93% for the VR group and control group, respectively. The median exercise adherence for VR and control groups was 78.89% (IQR 47.75%-142%) and 68.75% (IQR 38.57%-188%) during the initial 6 weeks, respectively; both groups had reduced exercise adherence, with rates decreasing to 77.22% (IQR 36.78%-104%) for the VR group and 62.08% (IQR 40.43%-166%) for the control group over the 12 weeks. The adherence rates in terms of bout time are shown in Table 2.

**Figure 1.**
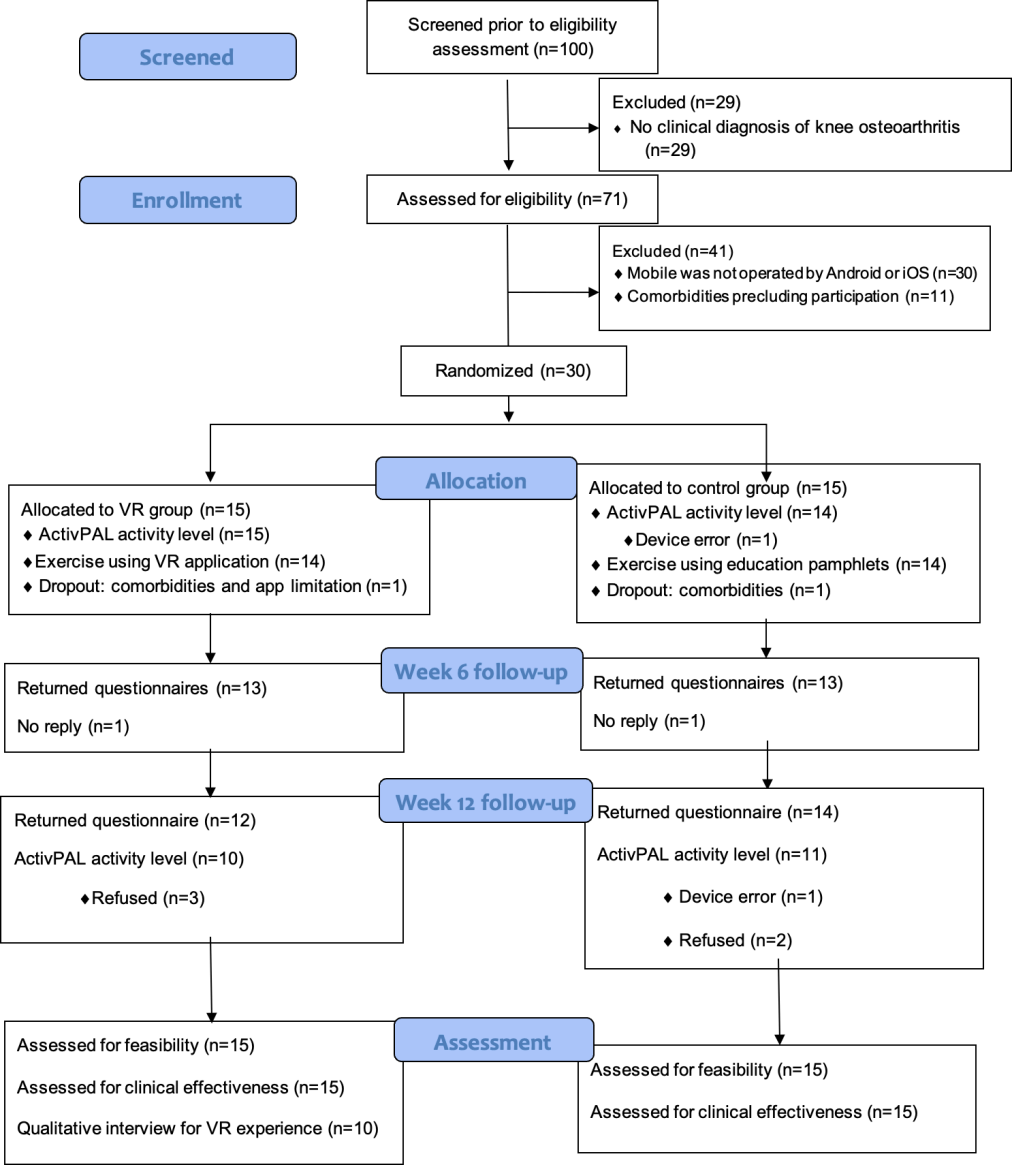
CONSORT diagram for reporting pilot and feasibility trials. CONSORT: Consolidated Standards of Reporting Trials; VR: virtual reality.

In total, 2 participants dropped out in the VRiKnee group and 1 in the control group, with a dropout rate of 13.3% and 6.7%, respectively. Reasons for dropout included hearing impairment (n=1) and worries for personal health (n=1) in the VR group and emotional distress (n=1) in the control group. Adverse events included 5 (33.3%) participants reporting cybersickness in the VR group. This study workflow is summarized in Figure 1.

**Table 2.** Primary outcomes between the intervention and control groups at week 12.

Primary outcomes	VRiKnee^a^	Control	*P* value^b^
Recruitment rate (%)	100	100	N/A^c^
Dropouts, n (%)	2 (13.3)	1 (6.67)	>.99
Response rate (%)	80	93	N/A
**Intervention adherence in terms of bout time measured by the TLFB-E** ^**d**^ **, median (IQR)**			
Weeks 1‐6	710 (429.75‐1275.75)	618.75 (347.13‐1692.75)	.78
Weeks 1‐12	1390 (662‐1877.8)	1117.5 (727.8‐2988)	.82
Cybersickness, n (%)	5 (33.3)	N/A	N/A

a VRiKnee: Immersive virtual reality–assisted lower limb strength training for knee osteoarthritis.

b Mann-Whitney test and intention-to-treat analyses were used for nonnormal continuous variables. Fisher exact was used for categorical variables .

c N/A: not applicable.

d TLFB-E: Timeline Followback for Exercise.

### Secondary Outcomes

At 12 weeks, no statistically significant between-group differences were detected for all secondary outcomes in this small pilot (Table 3). Yet, we detected a trend favoring the use of VRiKnee versus control in reducing the NRS (Cohen *d*=−0.084, *P*=.64) and WOMAC pain subscale (Cohen *d*=−0.089, *P*=.62) and improving the WOMAC stiffness subscale (Cohen *d*=−0.190, *P*=.29).

**Table 3.** Between-group differences of secondary outcomes at baseline, week 6, and week 12.

Variables	VRiKnee^a^ (n=15), mean (SD)	Control (n=15), mean (SD)	Mean difference between groups (95% CI, VRiKnee – control)^b^	*P* value	Cohen *d*
**NRS** ^c^					
Week 0	5.93 (1.86)	4.80 (1.49)	N/A^d^	N/A	N/A
Week 6	5.48 (1.74)	5.12 (1.22)	−0.04 (−1.31 to 1.22)	.95	−0.012
Week 12	4.78 (1.77)	4.56 (2.39)	−0.42 (−2.20 to 1.36)	.64	−0.084
**Total WOMAC** ^e^					
Week 0	902.93 (454.75)	751.97 (549.29)	N/A	N/A	N/A
Week 6	926.73 (544.51)	782.95 (444.68)	82.60 (−281.71 to 446.91)	.66	0.080
Week 12	791.30 (425.06)	742.36 (510.53)	−61.31 (−358.88 to 236.26)	.69	−0.073
**WOMAC pain**					
Week 0	189.53 (96.45)	163.87 (115.27)	N/A	N/A	N/A
Week 6	184.60 (117.51)	187.51 (102.71)	−14.20 (−99.41 to 71.02)	.74	−0.059
Week 12	160.16 (82.37)	160.86 (112.29)	−17.77 (−87.78 to 52.24)	.62	−0.089
**WOMAC stiffness**					
Week 0	91.27 (46.39)	65.60 (54.48)	N/A	N/A	N/A
Week 6	87.79 (51.21)	64.06 (40.28)	10.53 (−25.05 to 46.10)	.56	0.104
Week 12	68.79 (42.30)	69.04 (47.40)	−17.31 (−49.52 to 14.91)	.29	−0.190
**WOMAC function**					
Week 0	622.13 (339.36)	522.50 (393.99)	N/A	N/A	N/A
Week 6	653.83 (384.54)	530.50 (326.02)	89.66 (−165.68 to 345.00)	.49	0.124
Week 12	560.63 (312.72)	513.69 (364.69)	−22.55 (−232.23 to 187.14)	.83	−0.038
**EQ-VAS** ^f^					
Week 0	62.00 (19.62)	67.33 (17.20)	N/A	N/A	N/A
Week 6	67.58 (18.62)	70.83 (11.98)	0.45 (−10.35 to 11.25)	.94	0.014
Week 12	71.56 (12.79)	67.69 (17.42)	5.15 (−4.75 to 15.05)	.31	0.184
**ActivPAL – MET^g^ per day**					
Week 0	34.18 (1.24)	34.98 (1.35)	N/A	N/A	N/A
Week 12	34.35 (1.26)	34.91 (1.96)	−0.11 (−1.50 to 1.30)	.88	−0.146

a VRiKnee: immersive virtual reality–assisted lower limb strength training for knee osteoarthritis.

b Adjusting for baseline scores: analysis of covariance and intention-to-treat analyses were used. Covariates included intervention group, gender, age, comorbidities, metabolic equivalents of task, and variable at baseline.

c NRS: numeric rating scale.

d N/A: not applicable.

e WOMAC: Western Ontario and McMaster Universities Osteoarthritis Index.

f EQ-VAS: Euro-Qol-visual analogue scale.

g MET: Metabolic equivalents of task.

### Qualitative Outcomes

#### Overview

In total, 10 out of the 15 VR participants joined the qualitative interviews. Their background characteristics are summarized in Table 4. The duration of interviews lasted from 41 to 67 minutes. Further, 4 themes, 11 subthemes, and 16 quotes were generated upon analysis. The four themes included (1) embracing the use of VRiKnee, (2) facilitators of VRiKnee implementation, (3) barriers to VRiKnee adherence, and (4) suggestions for VR intervention development. The subthemes within each theme are presented in Tables 5-8.

**Table 4.** VRiKnee^a^ focus group participants characteristics at baseline, week 6, and week 12.

Participant	Age (years)	Gender	Chronic diseases (n)	MET^b^	NRS^c^ (score: range 0‐10)		WOMAC-pain^d^ (score: range 0‐500)		
					Baseline	Net change	Baseline	Week 6	Week 12
1	60	Female	5	32.69	8	−5	223	115	159
2	60	Male	2	33.42	3	+4	116	168	241
3	60	Female	2	35.38	7	−4	353	296	119
4	68	Male	2	33.86	5	−3	150	66	76
5	62	Male	3	33.24	5	−1	240	118	88
6	63	Female	0	32.20	8	−1	232	275	324
7	61	Female	0	34.66	6	−3	112	32	43
8	62	Female	2	34.15	6	−1	247	198	214
9	67	Female	1	36.49	6	−1	96	125.5	158.5
10	66	Male	2	34.04	5	0	97	68	N/A^e^

a VRiKnee: immersive virtual reality–assisted lower limb strength training for knee osteoarthritis.

b MET: metabolic equivalents of task.

c NRS: numeric rating scale.

d WOMAC-pain: Western Ontario and McMaster Universities Osteoarthritis Index pain subscale.

e N/A: not applicable (denotes missing data).

**Table 5.** Comparison between the quantitative and qualitative findings for embracing the use of VRiKnee^a^.

Quantitative findings	Qualitative findings (subthemes)	Conclusion
Our pilot study had a 100% recruitment rate. The recruitment periods of the 3 separate batches were completed in 2 days, 6 weeks, and 1.3 weeks, respectively.	*Craving technology despite challenges*: Participants expressed their interest in VR^b^ and all interviewees were positive to future use of advanced technology as a treatment modality.	Findings were complementary; VRiKnee was well-received by participants, with better exercise adherence and positive user experiences.
The participant retention rate was 87% and 93% for VRiKnee and control, respectively. The median exercise adherence for VRiKnee (77%, IQR 37%-104%) was higher than that for the control group (62%, IQR 40%-166%).	*Overcoming exercise inertia*: VRiKnee promoted a sense of achievement and satisfaction in users with exercise inertia by using gaming elements.	These findings support the above conclusions.

a VRiKnee: virtual reality–assisted lower limb strength training for knee osteoarthritis.

b VR: virtual reality.

**Table 6.** Comparison between the quantitative and qualitative findings for facilitators of VRiKnee^a^ implementation.

Quantitative findings	Qualitative findings (subthemes)	Conclusion
Although this small pilot RCT^b^ did not yield statistically significant results for both primary and secondary outcomes, we did observe a preliminary trend on the primary outcome of pain reduction on the NRS^c^ (*P*=.64; Cohen *d*=−0.084) and WOMAC-pain^d^ (*P*=.62, Cohen *d*=−0.089) at week 12.	*Improved knee osteoarthritis symptoms*: Participants who adhered to VRiKnee reported improvements in knee pain and function.	Findings were supplementary. Qualitative findings suggest that VRiKnee may offer potential benefits in managing knee osteoarthritis symptoms and enhancing exercise adherence, which might explain the observed positive, though statistically nonsignificant trends in some outcomes.
The median exercise adherence for VRiKnee (77%) was higher than control group (62%).	*Digital records enhancing exercise adherence*: Participants reported improved exercise compliance when using VRiKnee, with 5 participants noting that its record-keeping feature served as a reminder for them to exercise. Furthermore, 2 participants expressed their enhanced adherence, attributing to the competitive element of VRiKnee’s virtual scoreboard.	These findings support the above conclusions.

a VRiKnee: immersive virtual reality–assisted lower limb strength training for knee osteoarthritis.

b RCT: randomized controlled trial.

c NRS: numeric rating scale.

d WOMAC-pain: Western Ontario and McMaster Universities Osteoarthritis Index pain subscale.

**Table 7.** Comparison between the quantitative and qualitative findings for potential barriers to VRiKnee^a^ adherence.

Quantitative findings	Qualitative findings (subthemes)	Conclusion
Both groups had reduced exercise adherence over the 12 week period: VRiKnee group reduced from 78% to 56%, while control reduced from 69% to 48%.	*Boredom from repetition*: Our participants conveyed a sense of boredom resulting from stagnant audiovisual elements and repetitive exercise moves, which were perceived as too easy.	Findings were complementary. Quantitative results showed reduced exercise adherence with time, whereas the qualitative findings shed light on several challenges faced by participants during the VR^b^ intervention. Addressing these issues is essential to enhance the overall user experience and promote better adherence to VR-based interventions in the future.
N/A^c^	*Technological challenges*: Participants suggested that the use of electronic appliances required additional time and effort. They also expressed a growing sense of frustration during prolonged use, especially when persistent technological errors were encountered.	These findings support the above conclusions.
No relevant quantitative data were available.	*Inconvenience of HMD*^*d*^ *during exercise*: Participants reported that the use of HMD during exercise was inconvenient, attributing to its bulkiness when their mobile phones were installed and the sensation of feeling confined while wearing it over their eyes.	These findings support the above conclusions.
2 (13%) VR group participants reported cybersickness throughout the intervention.	*HMD-induced cybersickness*: 2 additional participants reported experiencing dizziness during the use of HMD in the focus group interviews; 1 participant reported experiencing blurred vision after prolonged use of HMD.	These findings support the above conclusions.

a VRiKnee: immersive virtual reality–assisted lower limb strength training for knee osteoarthritis.

b VR: virtual reality.

c N/A: not available.

d HMD: head-mounted display.

**Table 8.** Comparison between the quantitative and qualitative findings for suggestions for VR^a^ intervention development.

Quantitative findings	Qualitative findings (subthemes)	Conclusion
No relevant quantitative data were available.	*Enriching VR app’s audiovisual features*: Our participants suggested to increase the variety of scenes, interactive animations, and background music in different levels, which may contribute to a more engaging and enjoyable VR experience for users.	N/A^b^
No relevant quantitative data were available.	*Enhancing VR haptic interfaces*: Our participants suggested to improve the interfaces between the VR app and the motion sensor to ensure a more accurate digital record of the exercise moves.	N/A
No relevant quantitative data were available.	*Delivering VR experiences on 2D large screens*: Half of the participants expressed a preference in performing VR-assisted exercise using large “television screens” for an enhanced VR experience.	N/A

a VR: virtual reality.

b N/A: not available.

#### Theme 1: Embracing the Use of VRiKnee

##### Overview

Participants expressed a positive reception toward the implementation of VRiKnee in their treatment, acknowledging its potential as a valuable intervention.

##### Craving Technology Despite Challenges

Participants expressed a strong desire to incorporate advanced technology in managing their health conditions, despite facing certain challenges with the technology. The enthusiasm toward using VR for their health needs was unanimous among all participants.

*I really like it, actually, I am very fond of new technologies.*

[P8]

##### Overcoming Exercise Inertia

Participants highlighted the hedonic aspects of VR, such as visual stimulation and sound effects, had played a crucial role in overcoming exercise inertia. Even novice users, who initially hesitated to engage in physical activity, reported a newfound enjoyment in the VRiKnee experience, leading to a sense of accomplishment.

*It feels like a competition when there are consecutive exercise records in the app, having it done yesterday and today. It gives me a sense of fulfillment and a little desire to challenge myself.*

[P1]

### Theme 2: Facilitators of VRiKnee Implementation

#### Overview

Physiological improvements and enhanced exercise compliance were potential facilitators for the use of VRiKnee.

#### Improved Knee Osteoarthritis Symptoms

Participants reported experiencing tangible improvements in their knee osteoarthritis symptoms. The perceived benefits fostered a sense of hope and optimism, and encouraged engagement.

*After using it (VRiKnee), I’m not sure if it’s my perception, but I felt I had more strength in my legs.*

[P4]

#### Digital Records Enhancing Exercise Adherence

Participants highlighted the role of digital exercise records provided by VRiKnee in boosting their exercise adherence. The availability of these records served as helpful reminders, encouraging them to maintain regular exercise habits and thereby improving their compliance.

*I believe that using VRiKnee allows me to keep an exercise record. This helps me to keep a regular schedule and a more disciplined exercise habit.*

[P5]

### Theme 3: Potential Barriers to VRiKnee Adherence

#### Overview

Some barriers to VRiKnee adherence were also recognized.

#### Boredom From Repetition

Participants experienced declining interest and excitement in VRiKnee due to the repetitive nature of visual images and music.

*At first, the activities seemed interesting because they provided visual motivation. After a few months, it became a bit monotonous looking at the same screen.*

[P1]

*If you do it more frequently, the first level is no longer challenging.… If I could choose, I would start from level two.*

[P4]

*VR was exciting and fresh for the first and second day, but it becomes boring after a while. So I stopped using it after a week.*

[P10]

#### Technological Challenges

Participants faced challenges in navigating VRiKnee and operating the VR system, leading to potential frustration and reduced engagement.

*During these past few months, most of the time was spent to deal with technology, whether the sensors were working properly or not.*

[P1]

*I wasted a lot of time in dealing with this (VRiKnee). I realized that the battery power was always not enough; I also need to deal with the machine (HMD). Sometimes my phone screen froze, and my records were all gone, so I have to start all over again from level one and two.*

[P6]

*The screen turned upside down suddenly after you succeeded a few moves. Not only that, the images also kept spinning around! Why does it have to be like this? Can’t I just lift my legs on my own! I’m not interested in doing (VRiKnee) anymore.*

[P7]

#### Inconvenience of HMD During Exercise

Participants found the HMD to be heavy and uncomfortable to wear, and eventually abandoned the use of the HMD.

*Well, I felt that wearing the HMD on my head creates a lot of obstacles for exercising. The longer you use it, the more uncomfortable you feel.*

[P10]

#### HMD-Induced Cybersickness

Participants generally avoided using the HMD due to experiencing cybersickness, including dizziness and blurred vision. This discomfort negatively impacted their engagement with VRiKnee exercises, leading to reduced adherence to the intervention.

*Looking backwards with the HMD causes dizziness. You must turn around and focus on the focal point to proceed to the next step.*

[P5]

*If I use the HMD for long time and focus too much, I can’t read small fonts and even the large fonts!*

[P2]

### Theme 4: Suggestions for VR Intervention Development

#### Overview

Participants offered valuable insights and suggestions for the development of VR interventions.

#### Enriching VR App’s Audiovisual Features

Most participants expressed the need to increase the variation of scenes, interactive animations, and background music across different levels of exercise difficulty. They believe these would make VRiKnee more appealing and engaging, thereby motivating them to continue its use.

*It would be more appealing if the images changed after each level instead of being the same all the time. Additionally, the music variety could be improved, as it was monotonous! As we progress to higher levels, incorporating more lively and upbeat music would make it more engaging.*

[P10]

#### Enhancing VR Haptic Interfaces

Enhancing haptic interfaces would offer a more immersive and engaging experience for users, potentially increasing their motivation and adherence to the VRiKnee

*The interface (between the app and lower limb sensor) can be less complicated! And the sensor should be able to respond accurately!*

[P7]

#### Delivering VR Experiences on 2D Large Screens

Participants preferred to use 2D large screens which would provide a more comfortable and less intrusive experience, allowing them to engage with VRiKnee without the discomfort of wearing a HMD.

*For us, it’s hard to see with a small screen, so it’s better to use a larger screen.*

[P1]

### Integration of Quantitative and Qualitative Findings

Quantitative and qualitative findings are integrated and displayed in Tables 5-8.

## Discussion

### Principal Findings

In summary, VRiKnee was well-received by participants and provided positive user experience. The qualitative analysis revealed that VRiKnee showed promising potential for managing knee osteoarthritis symptoms and improving exercise adherence, which could explain the positive trends observed in some of the quantitative outcomes. However, the quantitative results indicated a decline in exercise adherence over time. The qualitative findings highlighted various challenges faced by the participants during the VR intervention, emphasizing the need to address these issues in order to enhance the overall user experience and foster better adherence to VR-based interventions in future applications.

### Comparison With Previous Studies and Implications for Research and Practice

Recently, a study conducted by Özlü et al [24] investigated the impact of disease-specific gamification through HMD on pain, physical function, and balance in participants with knee osteoarthritis. While this study population consisted of younger participants, with a mean age of 53 (SD 10.19) years, the researchers documented similar adverse effects such as cybersickness, nausea, and headache. Consequently, if immersive VR technology is to be employed in the management of knee osteoarthritis or other chronic diseases, the level of immersion must be carefully considered [56,57]. Interestingly, some participants suggested that using 2D screens may offer a more comfortable alternative, thereby prompting the exploration of the cave automatic virtual environment. This technology entails a cube-shaped room where screens project computer-generated images onto the walls, floor, and ceiling, allowing users to interact with the virtual environment via handheld controllers or trackers. Previous application of the cave automatic virtual environment system in training patients with Parkinson disease has yielded promising preliminary results [58].

Furthermore, Özlü et al [24] also observed that the beneficial effects of VR intervention diminished over time. Therefore, to sustain the positive effects of VR interventions, ensuring compliance is of utmost importance. Overcoming several obstacles during the design phase can facilitate this process. For instance, regular content updates can maintain player engagement and interest by introducing new levels, characters, game modes, or features that expand the gameplay experience. Additionally, community engagement is essential in fostering a strong player community. This can be achieved through encouraging player interaction, sharing of experiences, and providing feedback via online platforms such as forums and social media, as well as through in-game features such as leaderboards and multiplayer modes. Finally, continuous improvements based on player feedback will play a critical role in enhancing the intervention.

### Strengths and Limitations

This study’s strengths lie in its use of a mixed methods design, which contributes to a comprehensive understanding of VRiKnee, enhances validity through triangulation, provides valuable contextual insights, and generates practical implications on the design of future VR interventions. Further, 1 limitation of this study is the small sample size, which may restrict the trial’s ability to detect meaningful effect sizes.

### Conclusion

The feasibility and acceptability of VRiKnee in managing knee osteoarthritis have been demonstrated, suggesting its potential clinical efficacy. However, further confirmation through larger scale trials is necessary. VRiKnee has shown promise in enhancing exercise adherence, although a decline was observed over time. Participants faced challenges during the VR intervention, underscoring the need to address these barriers to improve adherence in future implementations.

## Supplementary material

10.2196/52563 Multimedia Appendix 1 This file displays the illustrations for immersive virtual reality–assisted lower limb strength training for knee osteoarthritis (VRiKnee).

10.2196/52563 Multimedia Appendix 2 Semistructured interview guide

10.2196/52563 Checklist 1 CONSORT-eHEALTH checklist (V 1.6.1).

## Abbreviations

HMD: head-mounted display
ITT: intention-to-treat
MET: metabolic equivalents of task
NRS: numeric rating scale
VR: virtual reality
VRiKnee: immersive virtual reality–assisted lower limb strength training for knee osteoarthritis
WOMAC: Western Ontario and McMaster Universities Osteoarthritis Index
