# Supplementary material for: Examining the Feasibility, Acceptability, and Preliminary Efficacy of an Immersive Virtual Reality–Assisted Lower Limb Strength Training for Knee Osteoarthritis: Mixed Methods Pilot Randomized Controlled Trial
Source: JMIR Serious Games. 2024 Sep 27;12:e52563. doi: 10.2196/52563 (PMC11451550; doi:10.2196/52563)

1. Smartphones were equipped with iOS/Android apps and used alongside an HMD (VR Shinecon 5.0), to create a virtual reality environment for exercise. A sensor was applied to detect lower limbs movement and record their exercise moves.


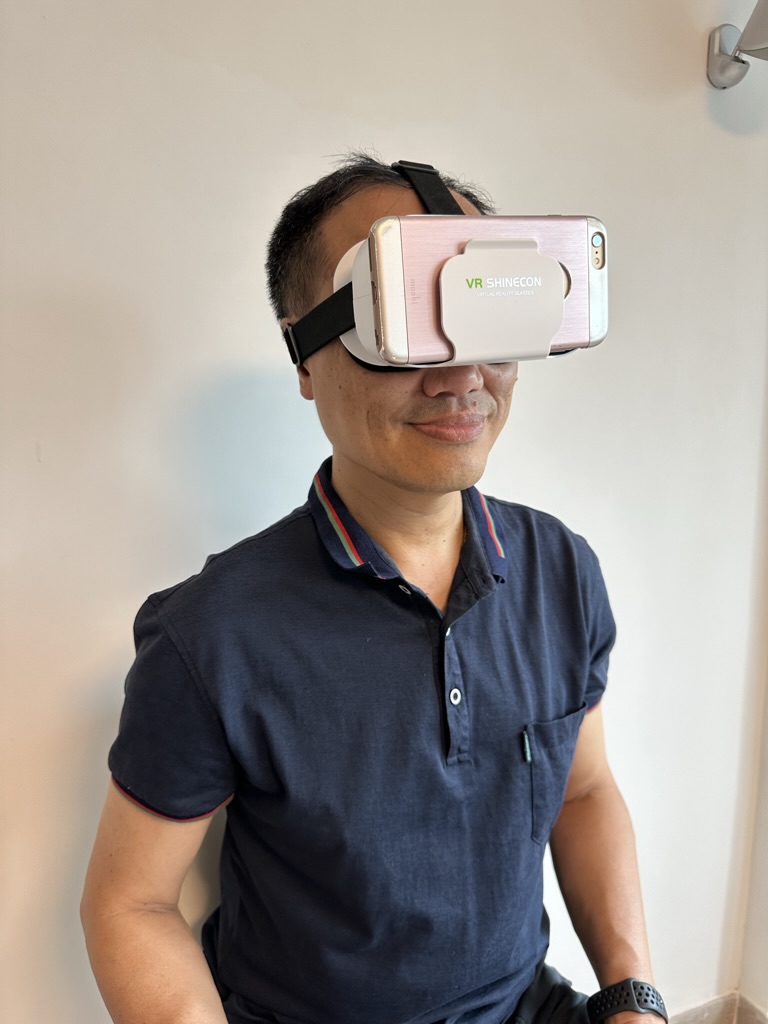

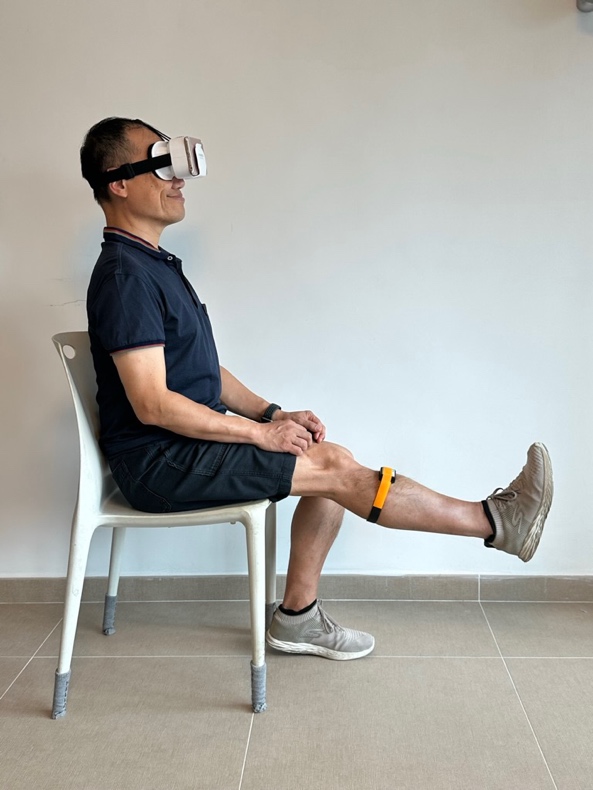


1. VRiKnee smartphone application:

A virtual environment was provided in the app, which incorporated ambient audio and guidance from an amateur coach. The images depict an immersive environment, where virtual flowers bloomed as a reward for successful exercise moves.


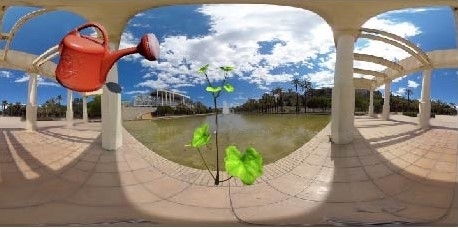

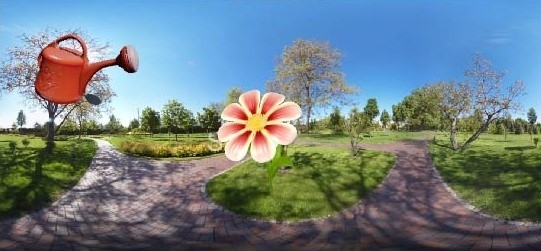


Additionally, the app included a timer for unlocking access to three levels of difficulty and featured a virtual scoreboard that recorded participants’ accumulated moves, total exercise time and game level.

1. The concentric quadriceps exercise


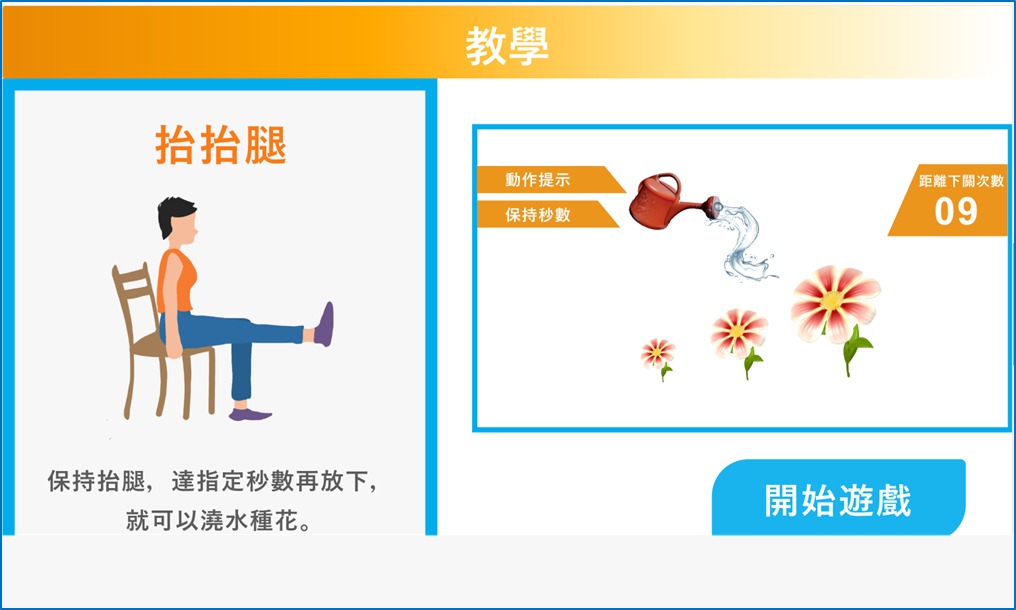


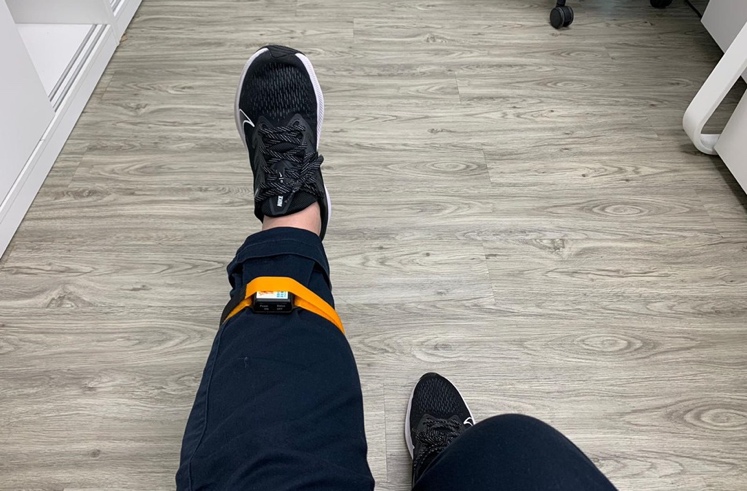


1. The isometric vastus medialis oblique (VMO) exercise


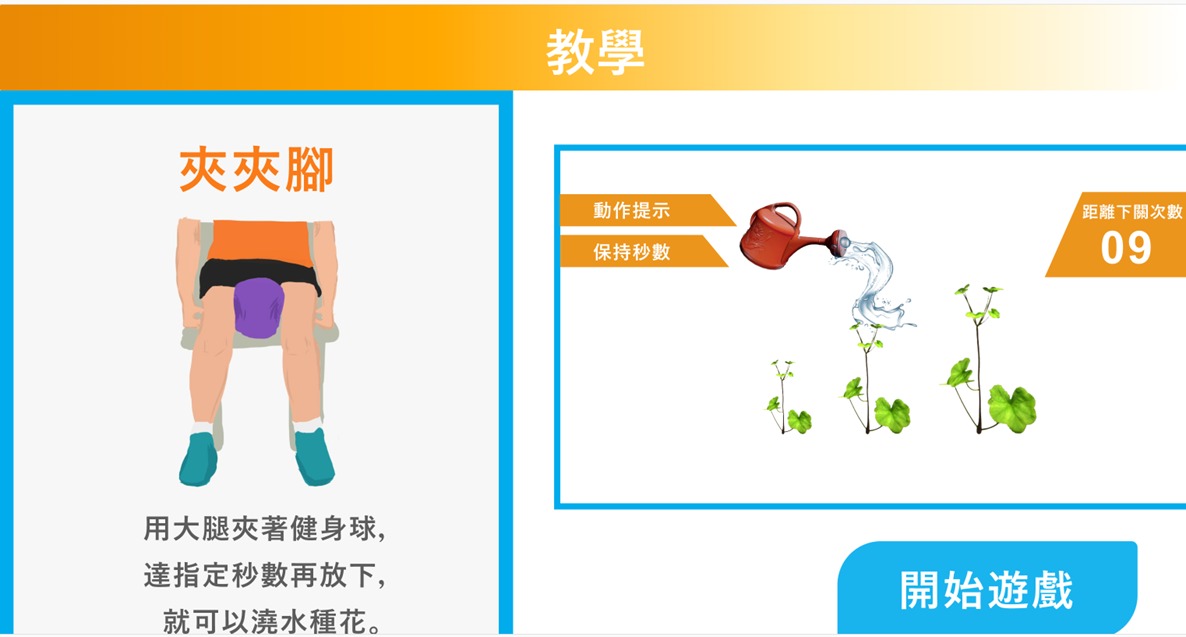


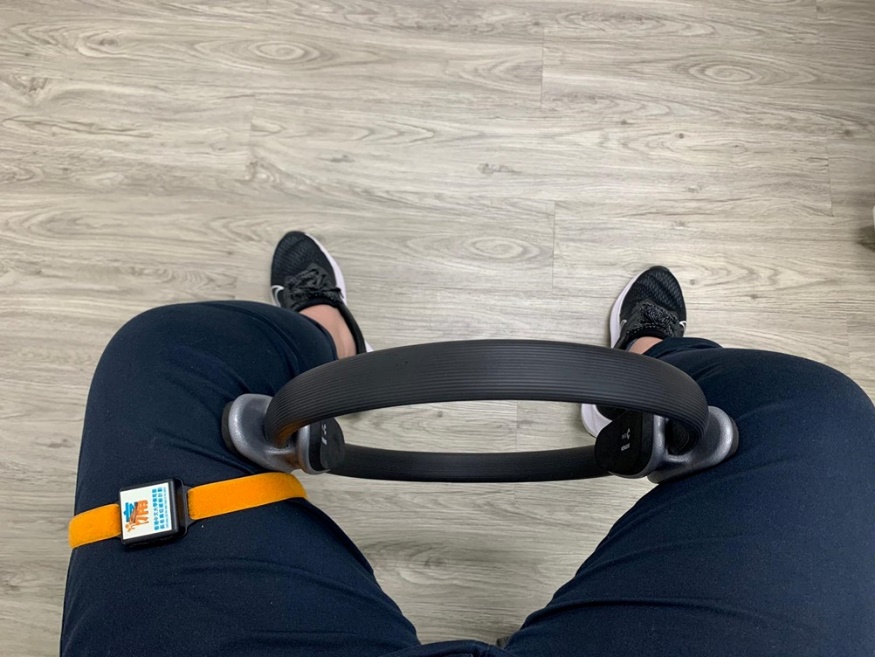


(c ) Virtual scoreboard


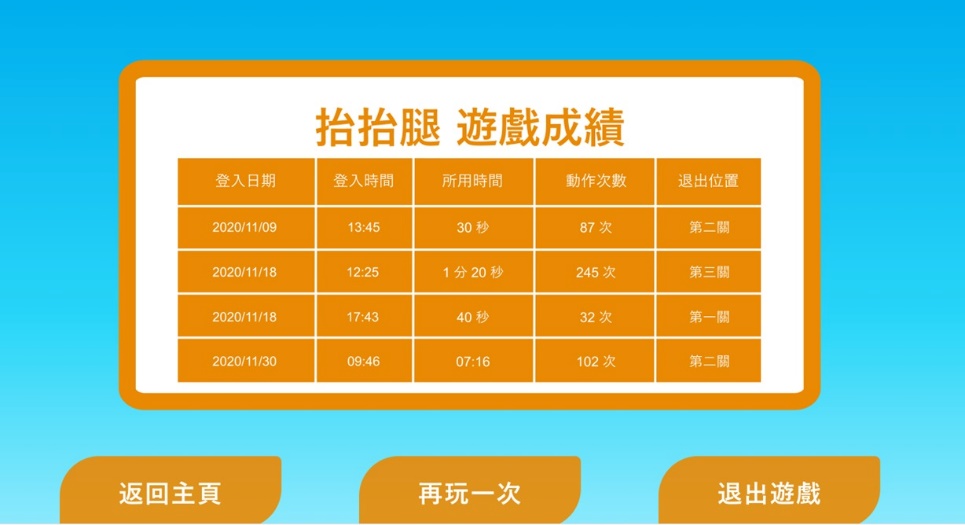

Supplement: Multimedia Appendix 1 [file games-v12-e52563-s001.docx]
