# Supplementary material for: Examining the Feasibility, Acceptability, and Preliminary Efficacy of an Immersive Virtual Reality–Assisted Lower Limb Strength Training for Knee Osteoarthritis: Mixed Methods Pilot Randomized Controlled Trial
Source: JMIR Serious Games. 2024 Sep 27;12:e52563. doi: 10.2196/52563 (PMC11451550; doi:10.2196/52563)
Supplement: Multimedia Appendix 2 [file games-v12-e52563-s002.docx]

1. Acceptance
2. How do you feel about VRiKnee?
3. How has VR influenced your engagement in lower limb strengthening exercises?
4. Share the benefits and drawbacks of incorporating VR into lower limb strengthening routine.
5. Perceived ease of use
   1. Can you describe the ease of use of VRiKnee?
   2. Did you encounter any challenges while using VRiKnee? How did you overcome them?
   3. Can you suggest any shortcomings of VRiKnee?
   4. What motivates you to use VRiKnee regularly/daily? Have you encountered any barriers to regular/daily use?
6. Perceived usefulness
   1. In what ways do you find VR useful in enhancing the lower limb strengthening exercises?
   2. What special features or functionalities of VRiKnee appeal to you?
   3. Have you observed/experienced any benefits resulting from the use of VRiKnee?
7. Intention to use
   1. Do you plan to continue using VRiKnee in the future?
   2. Which specific training component do you intend to continue practicing?
8. User experience
   1. Can you describe your overall experience using VRiKnee?
   2. What feedback or suggestions do you have for improving your VRiKnee experience? Are there any features or settings that you like to customize?
